# In Subfertile Couple, Abdominal Fat Loss in Men Is Associated with Improvement of Sperm Quality and Pregnancy: A Case-Series

**DOI:** 10.1371/journal.pone.0086300

**Published:** 2014-02-10

**Authors:** Céline Faure, Charlotte Dupont, Martin A. Baraibar, Romain Ladouce, Isabelle Cedrin-Durnerin, Jean Philippe Wolf, Rachel Lévy

**Affiliations:** 1 Service d'Histologie-Embryologie-Cytogénétique-CECOS, Hôpital Jean Verdier (AP-HP), Bondy, France; 2 Unité de Recherche en Epidémiologie Nutritionnelle, INSERM U557, INRA, CNAM, Université Paris 13, CRNH IdF, Bobigny, France; 3 INRA, UMR1198 Biologie du Développement et Reproduction, Jouy en Josas, France; 4 Laboratoire de Biologie Cellulaire du Vieillissement, Université Pierre et Marie Curie–Paris 6, Paris Cedex 05, France; 5 Service de Médecine de la Reproduction, Hôpital Jean Verdier, Assistance Publique—Hôpitaux de Paris, Université Paris XIII, Bondy, France; 6 Service d'Histologie-Embryologie-Biologie de la Reproduction, Hôpital Cochin, Université Paris Descartes, Paris, France; Inserm, France

## Abstract

**Background:**

The impact of overweight among men of reproductive-age may affect fertility. Abdominal fat, more than body mass index, is an indicator of higher metabolic risk, which seems to be involved in decreasing sperm quality.

This study aims to assess the relationship between abdominal fat and sperm DNA fragmentation and the effect of abdominal fat loss, among 6 men in subfertile couples.

**Methods:**

Sperm DNA fragmentation, abdominal fat and metabolic and hormonal profiles were measured in the 6 men before and after dietary advices. Seminal oxidative stress and antioxidant markers were determined.

**Results:**

After several months of a lifestyle program, all 6 men lost abdominal fat (patient 1: loss of 3 points of abdominal fat, patient 2: loss of 3 points, patient 3: loss of 2 points, patient 4: loss of 1 point, patient 5: loss of 4 points and patient 6: loss of 13 points). At the same time, their rate of sperm DNA fragmentation decreased: 9.5% vs 31%, 24% vs 43%, 18% vs 47%, 26.3% vs 66%, 25.4% vs 35% and 1.7% vs 25%. Also, an improvement in both metabolic (significant decrease in triglycerides and total cholesterol; p = 0.0139) and hormonal (significant increase in testosterone/oestradiol ratio; p = 0.0139) blood profiles was observed after following the lifestyle program. In seminal plasma, the amount of SOD2 has significantly increased (p = 0.0139) while in parallel carbonylated proteins have decreased. Furthermore, all spouses got pregnant. All pregnancies were brought to term.

**Conclusion:**

This study shows specifically that sperm DNA fragmentation among men in subfertile couples could be affected by abdominal fat, but improvement of lifestyle factor may correct this alteration. The effect of specific abdominal fat loss on sperm quality needs further investigation. The reduction of oxidative stress may be a contributing factor.

## Introduction

Infertility affects about 15% of couples in reproductive age. A male factor, usually of unknown origin, is involved in 40% of cases [1].

The high prevalence of overweight and obesity is significantly contributing to the overall burden of diseases worldwide, including infertility. In women, deleterious effects of obesity on reproductive functions are well documented and extensively published, dealing especially with higher frequency of ovulatory disorders and menstrual irregularities [2], [3]. Within this context, abdominal fat accumulation contributes to reproductive dysfunction [4], [5].

Although there are still controversies about the effect on males, we recently show an increased risk of azoospermia or oligozoospermia in male with high body mass index (BMI) [6], [7]. Overweight or obesity may also alter sperm function, as an increase in sperm DNA fragmentation has been observed [8], [9], [10], [11]. Abdominal fat accumulation appears to be a better indicator of high risk for developing an abnormal metabolic profile than body mass index. A recent study showed that central adiposity, defined by a high waist circumference [12], negatively affects sperm concentration and total motile sperm count.

Indeed, adipose tissue accumulation is associated with increased oxidative stress, one of the potential mechanisms to explain sperm damages in obese patients [13], [14]. Decreased seminal plasma antioxidant and increased ROS production can be responsible for idiopathic male infertility [15].

Protein carbonyls, formed by a variety of oxidative mechanisms, are sensitive indices and the most general and commonly used biomarker of oxidative injury [16]. By contrast, it is interesting to evaluate SOD2, a ROS scavenger.

In women, abdominal fat loss results in an improvement of fertility [17], [18]. In men, only one publication is available and suggests an improvement of sperm parameters following weight loss [19].

The aim of this report is to investigate the association between abdominal fat loss in subfertile men and sperm quality. We also report the achievement of pregnancy following dietary advices in six subfertile men and analysis improvement of their sperm parameters, seminal antioxidant and oxidative stress markers and metabolic and hormonal profiles after abdominal fat loss.

## Materials and Methods

### Subjects

A cohort of couples attending an infertility centre for a primary idiopathic infertility (n = 36) was recruited for the ALIFERT study. ALIFERT study's criteria imply that patients do not have or did not have a metabolic disease (dyslipidemia, high blood pressure, hyperglycemia, diabetes...) nor a digestive disease. A sub-cohort of men was asked to participate to a personalized dietary program, including individualized nutritional advice aiming at reducing intra-abdominal fat and increasing exercise.

In this sub-cohort, we selected non smoking men, presenting with a percentage of sperm DNA fragmentation ≥25%, with abdominal fat ≥4 as measured by impedancemetry (see below) and still in a facto relationship.

**Figure 1:**
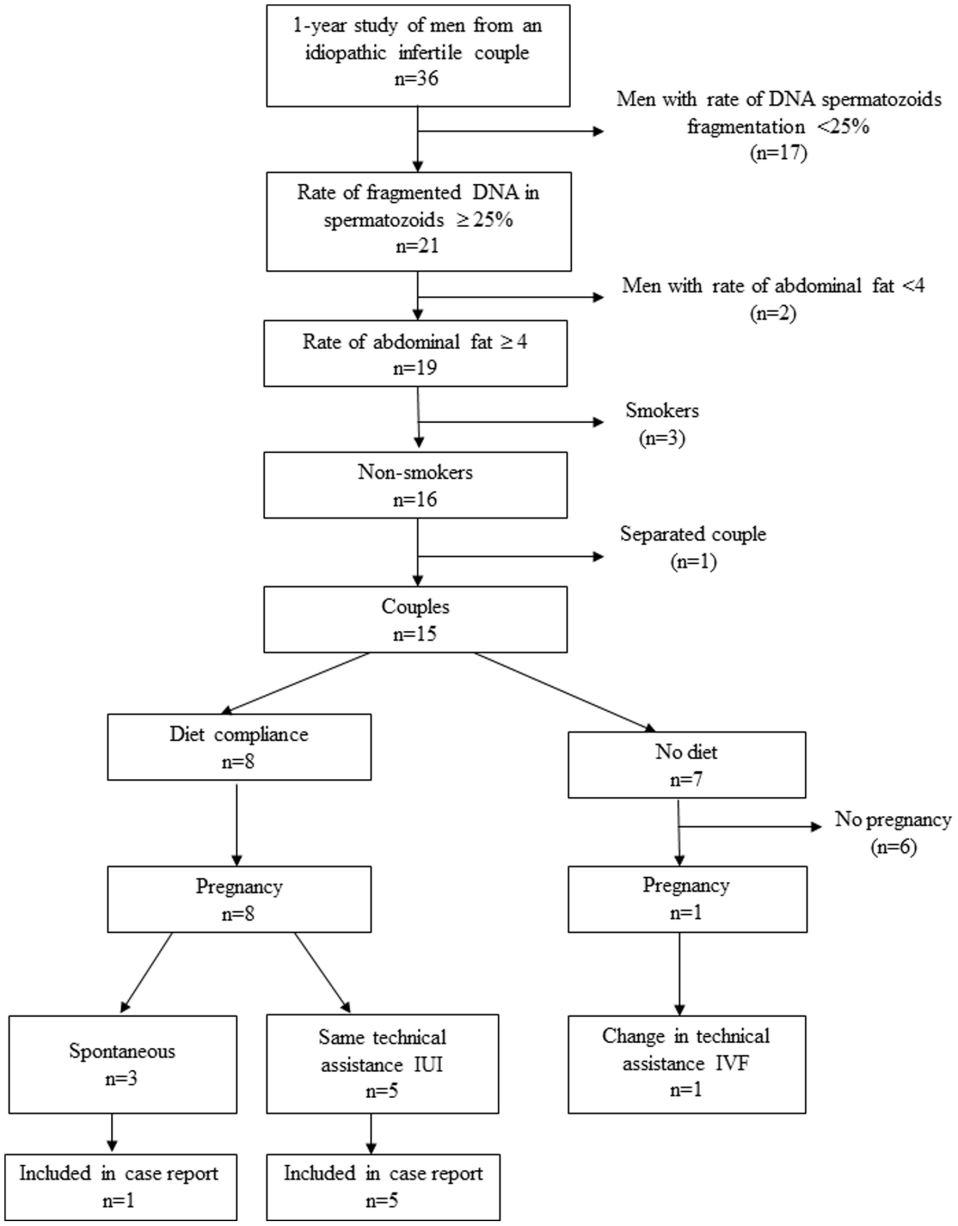
Design of the study.

Out of 15 men responding to the above criteria, 8 accepted to follow the dietary program. Among them, only 6 agreed to provide samples before and after the intervention (Figure 1).

Before and after the dietary program, anthropometric measures, abdominal fat, sperm parameters and sperm DNA fragmentation were evaluated. Hormonal and metabolic profiles could be obtained. Occurrence and outcome of pregnancies, obtained either spontaneously or following Assisted Reproductive Technology, were recorded.

The men received no incentives and participation was conditional to written and informed consent. The local ethics committee (Comité de Protection des Personnes) approved the study.

### Anthropometric assessment

Height was measured to the nearest 5 mm, without shoes, using a metric, by the same trained investigator. Waist circumference was measured at the narrowest point between the lower border of the ribs and the iliac crest. Weight and body composition were evaluated using the Tanita BC-420MA Analyzer. The bioelectrical impedance measurement combines a digital scale with stainless steel pressure-contact foot-pad electrodes for standing impedance and body weight measurements [20]. Details of the validation and performance characteristics of this bioimpedance analysis model have been reported previously [21]. The bioimpedance supplies a level of visceral fat: the purpose is to be as close to 1; rating from 1 to 4 indicates a low level of visceral fat; rating 5 to 12 indicates a high level of visceral fat; >12 indicates a very excessive level.

### Semen analysis

Sperm samples were collected at the laboratory after 3–5 days of sexual abstinence. After semen liquefaction, semen analysis was performed according to World Health Organization guidelines (WHO, 2010) assessing semen volume, sperm concentration, sperm motility. Total sperm count (TSC) was calculated.

### Determination of sperm DNA integrity

To evaluate sperm nuclear DNA integrity, the TUNEL (Terminal Uridine Nick End Labeling) technique [22], [23] was performed on semen samples using In Situ Cell Death Detection Kit (In situ Cell Death Detection kit, Fluorescein, Roche Applied Science). Briefly, after trypsinization, spermatozoa were fixed in Carnoy solution (2∶1 methanol/acetic acid) and stored at −20°C. Spermatozoon pellets were permeabilised for 20 minutes with 0,1% Triton X-100 in sodium citrate solution and washed with PBS. Then cells were incubated with dUTP FITC-labelled and terminal deoxyribonucleotidyl tranferase (TdT) (TUNEL solution). The positive control sample was treated with 100 µl of DNase (0,5 mM) for one hour at 37°C before incubation with the TUNEL solution and the TdT enzyme was omitted for the negative control. Cells were then washed twice in PBS and spread out over slides. Slides were dried at room temperature in the dark and DAPI solution was added over the spermatozoa. Slides were examined using fluorescence microscopy. At least 200 spermatozoa were counted and total sperm DNA fragmentation rate was calculated as the number of FITC-positive cells from the total number of sperm nuclei (labelled with DAPI). Two investigators blinded to the exposure and other covariates performed twice the analyses.

### Seminal markers assays

Liquefied semen was centrifuged to separate sperm from seminal plasma. Seminal plasma was immediately stored in aliquots at −80°C for further analysis without freezing–thawing.

### Superoxide dismutase

SOD2 was quantified by ELISA using the “Human Superoxyde Dismutase 2 ELISA” kit (Abfrontier) according to manufacturer's instructions.

### Detection of carbonylated proteins

Carbonylated proteins were labeled with CyDyeTM hydrazides (GE Healthcare) as described previously [24]. Briefly, samples were homogenized using a lysis buffer (10 mM tris-HCl (pH 7.4), 8 M urea, 2 M thiourea, 4% (w/v) CHAPS, 10 mM dithiothreitol (DTT)) and clarified by centrifugation. Protein quantification was performed by the Bradford method (Bio-Rad protein assay), using BSA as standard. Carbonylated proteins were labeled with Cy5 hydrazides (GE Healthcare) and total proteins were precipitated using the 2-D Clean-Up kit (GE Healthcare), following the manufacturer instructions. Protein precipitates were resuspended in loading buffer and separated by SDS-PAGE (4-20%). Total proteins were post-stained with ProteinGOLD (gel company). Fluorescent scanning was performed using Ettan Dalt system (GE Healthcare) at excitation and emission wavelengths of 635/680 for the Cy5 hydrazide and 390/595 for total proteins, respectively. Semiquantification of carbonylated proteins were performed on digitalized images by densitometric analysis using total protein staining as loading control.

### Dietary program

All participants received individualized dietary advice by a nutritionist after a complete nutritional assessment. Program was based on a healthy, balanced diet, aiming for reduction in abdominal fat according to the French national nutrition and health programme. They were encouraged to practice 1 h of weekly exercise. Spouses did not change neither their diet nor their lifestyle.

### Blood collection

Blood samples were used to estimate fasting glucose, lipid profile (triglycerides (TG), total cholesterol, HDL and LDL) and for sex hormone assays (FSH, LH, oestradiol and testosterone). The testosterone/oestradiol (T/E) ratio was calculated.

### Statistical analysis

To determine whether there is an overall difference in pre- and post-intervention, anthropometric and sperm parameters, metabolic and sex hormones values were compared between the pre-test and the post-test using the Wilcoxon test. Value of 0.05 or less was considered statistically significant.

## Results

**Figure 2:**
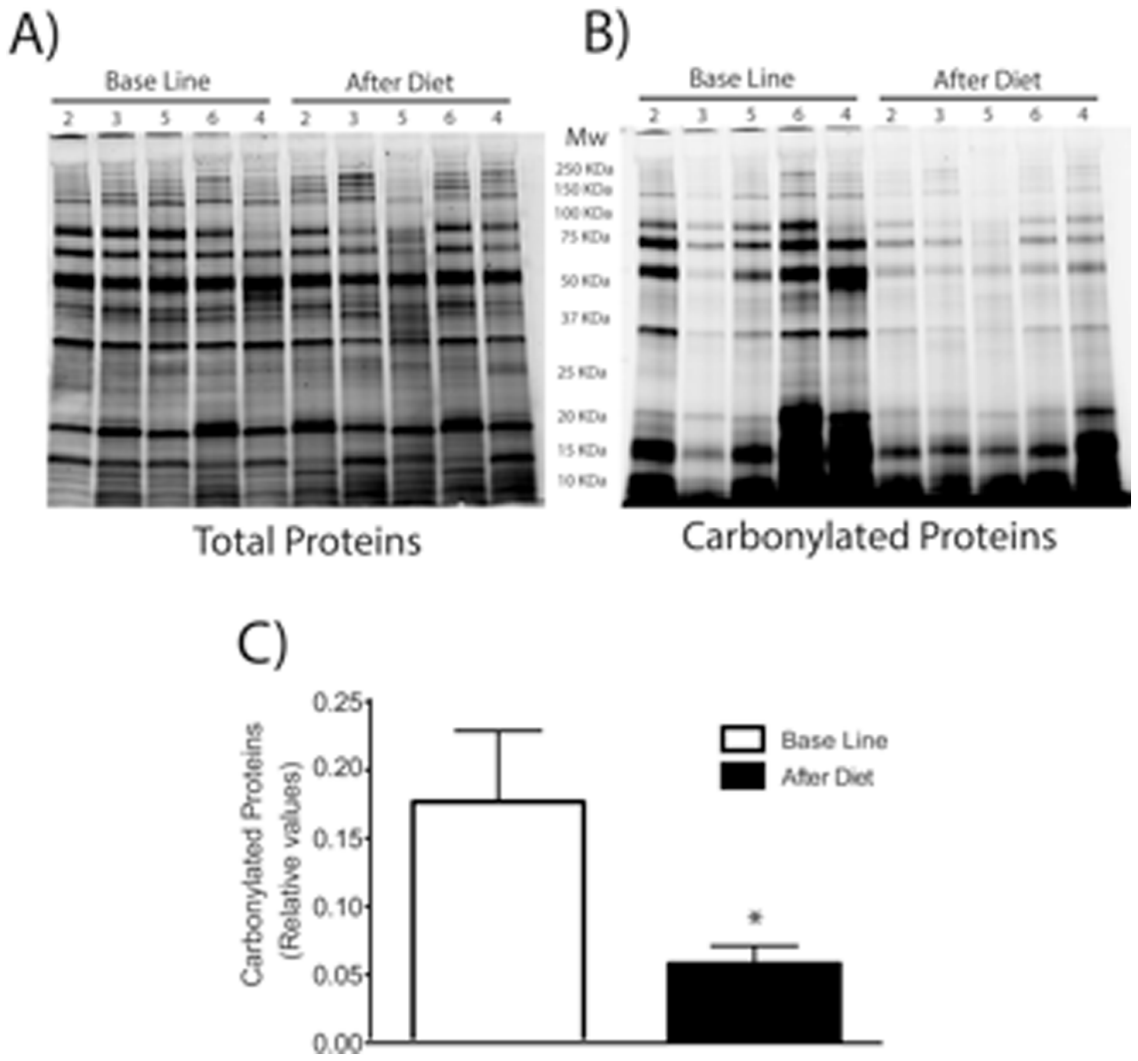
Oxidized damaged proteins before (Basal Line) and after treatment (After Diet). SDS-PAGE (4–20%) pattern of total proteins post-stained with ProteinGOLDTM (A), or carbonylated proteins pre-labeled with C5Hz (B). Semiquantification of carbonylated proteins were performed by densitometric analysis, expressed as relative values and shown as mean±S.D (n = 5) and analyzed using Student's t-test; * P = 0.06 (C).

The characteristics of the six patients at baseline and after diet are presented in Table 1 and Figure 2.

**Table 1:** Anthropometric parameters, semen characteristics, seminal antioxidant markers, metabolic and hormonal profiles and pregnancy outcome at baseline and after dietary advices.

	Patient 1		Patient 2		Patient 3		Patient 4		Patient 5		Patient 6		Wilcoxon test
	baseline	after diet	baseline	after diet	baseline	after diet	baseline	after diet	baseline	after diet	baseline	after diet	signification (alpha s)
**Anthropometry**													
age (years)	30		29		33		28		27		44		
height (m)	1.86		1.82		1.89		1.73		1.88		1.89		
weight (kg)	87.5	86.2	93.7	87.7	160.6	146.7	79.5	78.4	96.2	94.7	111.6	109	0.0139*
BMI (kg/m^2^)	25.3	24.9	28.3	26.5	44.9	41.1	26.6	26.2	27.2	26.8	31.2	30.7	0.0139*
waist circunference (cm)	103	90	90	89	142	121	83	80	96	94	125	114	0.0139*
intra-abdominal fat	6	3	4	3	26	13	6	3	7	5	13	9	0.0139*
**Sperm analysis**													
** **total sperm count (M)	28.4	16.9	76.2	31.9	35.6	87	74	199.6	29	176.6	21.9	32	0.1244
progressive sperm motility (%)	40	40	30	30	8	10	40	25	25	45	8	35	0.1367
normal spermatozoa (%)	31	27	14	15	11	21	26	19	7	14	11	16	0.2068
sperm fragmentation (%)	31	9.5	66	26.3	25	1.7	43	24	47	18	35	25.4	0.0139*
**Seminal plasma analysis**													
superoxide dismutase protein 2 (pg/ml)	1191.5	2741.5	809	3621.5	1819	3311.5	4191.5	>4399	754	3499	2939	4484	0.0139*
**Biological parameters**													
** **fasting glucose (g/l)	0.9	0.83	0.82	1.01	0.94	0.92	0.88	0.87	0.95	0.97	0.97	0.83	0.3766
triglycerides (g/l)	0.82	0.7	0.89	0.59	1.87	1.39	1.02	0.7	1.21	1.1	3.07	2.19	0.0139*
total cholesterol (g/l)	2.01	1.72	2.2	1.64	1.75	1.62	2.01	1.69	1.86	1.64	3.17	2.63	0.0139*
HDL (g/l)	0.46	0.37	0.52	0.37	0.43	0.54	0.47	0.38	0.51	0.5	0.62	0.5	0.0865
LDL (g/l)	1.4	1.21	1.51	0.95	0.96	0.97	1.35	1.17	1.11	1.52	2.11	1.52	0.1244
HDL/LDL ratio	0.33	0.31	0.34	0.39	0.45	0.56	0.35	0.32	0.46	0.33	0.29	0.33	
FSH (UI/l)	4.6	3.2	4.6	4.5	3.6	3.2	2.1	2.3	45	4.1	2.6	2.3	0.0374*
LH (UI/l)	5.3	2.7	4.9	4.8	3.9	2.7	3	2.7	3.3	2.9	2.3	2.7	0.0865
oestradiol (pg/ml)	51.7	36	37.4	22	34.6	32.8	32.8	26	30.3	28	26.7	18.1	
testosterone (ng/ml)	2.31	5.2	1.81	5.52	1.83	2.78	5.07	5.82	2.07	3.8	1.53	2.06	
testo/oestra ratio	44.6	144.4	48.4	250.9	52.9	87.76	154.6	223.8	68.3	135.7	57.3	113.8	0.0139*
**Assisted reproductive treatment**	1 stim 1 IUI	1 IUI	1 stim	1 IUI	2 stim 1 IUI	1 IUI	1 stim 3 IUI	1 IUI	2 stim	None	3 IUI	1 IUI	
**Outcome**	No pregnancy	Live birth	No pregnancy	Live birth	No pregnancy	Live birth	No pregnancy	Live birth	No pregnancy	Live birth	No pregnancy	Live birth	

### Patient 1

Patient 1 was a 30-year-old man with a 21-month history of primary idiopathic infertility. His partner was 30 years old and had a BMI at 26.3 kg/m2; her ovulatory status, hormonal profile and pelvic examination were within normal values.

At the time of the visit, male BMI was 25.3 kg/m2, abdominal fat was 6, sperm DNA fragmentation rate was 31% and sperm parameters were: TSC 28.4×10^6^, progressive motility 40% and 31% normal spermatozoa. In seminal plasma concentration of SOD2 was 1191.5 pg/ml. Ratio of sex hormones was: T/E 44.6 and lipid profile: TG 0.82 g/l and total cholesterol 2.01 g/l. The couple had previously benefited from one ovarian stimulation, and one intrauterine insemination (IUI) without any pregnancy.

After 3 months on the dietary program, a pregnancy was obtained following the second IUI. At this time, male BMI was 24.9 kg/m2, abdominal fat was at 3, sperm DNA fragmentation rate was 9.5% and sperm parameters were: TSC 16.9×10^6^, progressive motility 40% and 27% normal spermatozoa. In seminal plasma concentration of SOD2 was 2741.5 pg/ml. Ratio of sex hormones was: T/E 144.4 and lipid profile: TG 0.7 g/l and total cholesterol 1.72 g/l.

### Patient 2

Patient 2 was a 28-year-old man with a 36-months history of primary idiopathic infertility. His partner was 31 years old and had a BMI at 19 kg/m2; her ovulatory status, hormonal profile and pelvic examination were within normal values.

At the time of the visit, male BMI was 26.6 kg/m2, abdominal fat was at 6, sperm DNA fragmentation rate was 43% and sperm parameters were: TSC 74×10^6^, progressive motility 40% and 26% normal spermatozoa. In seminal plasma concentration of SOD2 was 809 pg/ml. Ratio of carbonylated proteins to total proteins was 0.16. Ratio of sex hormones was: T/E 154.6 and lipid profile: TG 1.02 g/l and total cholesterol 2.01 g/l. The couple had previously benefited from one ovarian stimulation cycle and three IUI without any pregnancy.

After 4 months on the dietary program, a pregnancy was obtained following the fourth IUI. At this time, male BMI was 26.2 kg/m2, abdominal fat was at 3, sperm DNA fragmentation rate was 24% and sperm parameters were: TSC 199.6×10^6^, progressive motility 25% and 19% normal spermatozoa. In seminal plasma concentration of SOD2 was 3621.5 pg/ml. Ratio of carbonylated proteins to total proteins was 0.06. Ratio of sex hormones was: T/E 223.8 and lipid profile: TG 0.7 g/l and total cholesterol 1.69 g/l.

### Patient 3

Patient 3 was a 27-year-old man with an 18-months history of primary idiopathic infertility. His partner was 31 years old and had a BMI at 27.7 kg/m2; her ovulatory status, hormonal profile and pelvic examination were within normal values.

At the time of the visit, male BMI was 27.2 kg/m2, abdominal fat was at 7, sperm DNA fragmentation rate was 47% and sperm parameters were: TSC 29×10^6^, progressive motility 25% and 7% normal spermatozoa. In seminal plasma concentration of SOD2 was 1819 pg/ml. Ratio of carbonylated proteins to total proteins was 0.04. Ratio of sex hormones was: T/E 68.3 and lipid profile: TG 1.21 g/l and total cholesterol 1.86 g/l. The couple had previously benefited from 2 ovarian stimulations without any pregnancy.

After 5 months on the dietary program, a spontaneous pregnancy was obtained. At this time, male BMI was 26.8 kg/m2, abdominal fat was at 5, sperm DNA fragmentation rate was 18% and sperm parameters were: TSC 176.6×10^6^, progressive motility 45% and 14% normal spermatozoa. In seminal plasma concentration of SOD2 was 3311.5 pg/ml. Ratio of carbonylated proteins to total proteins was 0.05. Ratio of sex hormones was: T/E 135.7 and lipid profile: TG 1.1 g/l and total cholesterol 1.64 g/l.

### Patient 4

Patient 4 was a 29-year-old man with a 25-month history of primary idiopathic infertility. His partner was 29 years old and had a BMI at 25.7 kg/m2; her ovulatory status, hormonal profile and pelvic examination were within normal values.

At the time of the visit, male BMI was 28.3 kg/m2, abdominal fat was at 4, DNA fragmentation rate was 66% and sperm parameters were: TSC 76.2×10^6^, progressive motility 30% and 14% normal spermatozoa. In seminal plasma concentration of SOD2 was 4191.5 pg/ml. Ratio of carbonylated proteins to total proteins was 0.26. Ratio of sex hormones was: T/E 48.4 and lipid profile: TG 0.89 g/l and total cholesterol 2.2 g/l. The couple had previously benefited from one ovarian stimulation cycle without any pregnancy.

After 4.5 months on the dietary program, a pregnancy was obtained following the first IUI. At this time, male BMI was 26.5 kg/m2, abdominal fat was at 3, sperm DNA fragmentation rate was 26.3% and sperm parameters were: TSC 31.9×10^6^, progressive motility 30% and 15% normal spermatozoa. In seminal plasma concentration of SOD2 was more than 4399 pg/ml. Ratio of carbonylated proteins to total proteins was 0.12. Ratio of sex hormones was: T/E 250.9 and lipid profile: TG 0.59 g/l and total cholesterol 1.64 g/l.

### Patient 5

Patient 5 was a 44-year-old man with a 36-months history of primary idiopathic infertility. His partner was 37 years old and had a BMI at 28.5 kg/m2; her ovulatory status, hormonal profile and pelvic examination were normal.

At the time of the visit, male BMI was 31.2 kg/m2, abdominal fat was at 13, sperm DNA fragmentation rate was 35% and sperm parameters were: TSC 21.9×10^6^, progressive motility 8% and 11% normal spermatozoa. In seminal plasma concentration of SOD2 was 754 pg/ml. Ratio of carbonylated proteins to total proteins was 0.10. Ratio of sex hormones was: T/E 57.3 and lipid profile: TG 3.07 g/l and total cholesterol 3.17 g/l. The couple had previously benefited from 3 IUI without any pregnancy.

After 8 months on the dietary program, a pregnancy was obtained following the fourth IUI. At this time, male BMI was 30.7 kg/m2, abdominal fat was at 9, sperm DNA fragmentation rate was 25.4% and sperm parameters were: TSC 32×10^6^, progressive motility 35% and 16% normal spermatozoa. In seminal plasma concentration of SOD2 was 3499 pg/ml. Ratio of carbonylated proteins to total proteins was 0.04. Ratio of sex hormones was: T/E 113.8 and lipid profile: TG 2.19 g/l and total cholesterol 2.63 g/l.

### Patient 6

Patient 6 was a 33-year-old man with a 24-months history of primary idiopathic infertility. His partner was 31 years old and had a BMI at 29.6 kg/m2; her ovulatory status, hormonal profile and pelvic examination were within normal values.

At the time of the visit, male BMI was 44.9 kg/m2, abdominal fat was at 26, sperm DNA fragmentation rate was 25% and sperm parameters were: TSC 35.6×10^6^, progressive motility 8% and 11% normal spermatozoa. In seminal plasma concentration of SOD2 was 2939 pg/ml. Ratio of carbonylated proteins to total proteins was 0.32. Ratio of sex hormones was: T/E 52.9 and lipid profile: TG 1.87 g/l and total cholesterol 1.75 g/l. The couple had previously benefited from 2 ovarian stimulations and one IUI without any pregnancy.

After 8 months on the dietary program, a pregnancy was obtained after the third IUI. At this time, male BMI was 41.1 kg/m2, abdominal fat was at 13, sperm DNA fragmentation rate was 1.7% and sperm parameters were: TSC 87×10^6^, progressive motility 10% and 21% normal spermatozoa. In seminal plasma concentration of SOD2 was 4484 pg/ml. Ratio of carbonylated proteins to total proteins was 0.06. Ratio of sex hormones was: T/E 87.8 and lipid profile: TG 1.39 g/l and total cholesterol 1.62 g/l.

General comment: All participants lost abdominal fat (in terms of body composition and waist circumference) with no change of BMI categories after following the lifestyle program. Furthermore, there was no substantial change in conventional sperm parameters, whereas a significant improvement of sperm DNA integrity was observed for all patients (p = 0.0139). In seminal plasma we observed a large increase of SOD2 protein and a decrease of carbonylated proteins.

At baseline, none had impaired fasting glucose while increased triglycerides were observed for the two obese men with concomitant increase in total cholesterol for one of them. All men except one had decreased levels of testosterone. An improvement in both metabolic (significant decrease in triglycerides and total cholesterol; p = 0.0139) and hormonal (significant increase in testosterone/oestradiol ratio; p = 0.0139) profiles was observed after following the dietary/lifestyle program.

All female partners achieved pregnancy and delivered a healthy child. Delivery and neonatal characteristics are presented in Table 2.

**Table 2:** Delivery and neonatal characteristics.

	Patient 1	Patient 2	Patient 3	Patient 4	Patient 5	Patient 6
amenorrhea weeks	39.5	40	40	37	38	41
baby weight (kg)	3230	3070	3580	2750	3300	3750
size (cm)	47	49	53	45	47	50
APGAR	10	10	10	10	10	10
sex	girl	girl	boy	girl	girl	girl

## Discussion

Although some studies failed to link BMI and semen analyses [25], [26], [27], most of them reported a detrimental impact of male BMI on sperm parameters, notably a decrease in sperm concentration [6], [7], [9], [10]. Consequently, the possibility to improve semen quality through weight reduction was also considered. Improvement of hormonal status was mainly observed after weight loss [28], [29]. Furthermore, a positive impact on semen parameters was observed by Hakonsen et al. [30].

However, abdominal fat, regardless of BMI, is a reliable predictor of individual risk of comorbidities such as metabolic syndrome [31], [32], [33]. Likewise, abdominal fat could be a good indicator to assess the risk of altered semen parameters. Until now, only 2 studies have shown a negative impact of abdominal fat (measured by waist circumference) on conventional semen parameters [12], [34] and none have assessed the impact of abdominal fat loss on male reproductive function.

Our report suggests, for the first time, that reduction of abdominal fat is associated with sperm DNA integrity improvement, regardless of BMI. Fat loss was also associated with an improvement of metabolic and sex steroid profiles. All 8 non smoking patients who followed the dietary program (6 agreed to provide samples after the intervention and 2 did not) have lost weight and their WC have decreased. Of the 7 patients who wished not to participate on the dietary program, no anthropometric change was observed.

Sperm DNA integrity is essential for a successful pregnancy [35]. High rates of sperm DNA fragmentation are associated with lower implantation rates and higher miscarriage rates [36], [37]. In this report, a successful pregnancy was obtained for the six couples after sperm DNA integrity improvement. The 2 other couples whose male partners followed the dietary program conceived healthy children. After one year, of the 7 patients who did not participate, six could not achieve pregnancy and one conceived a child after IVF. Information about sperm quality was not available.

Abdominal fat is associated with increased oxidative stress [38], [39]. Although, seminal plasma and sperm contain antioxidants, these defense systems can be overwhelmed [40]. Oxidative stress is known to impact male fertility. ROS (reactive oxygen species) can alter sperm membrane and sperm nucleus leading to sperm DNA damage [41], [42], [43]. Decreased SOD might be involved in abnormal semen quality [44]. Several studies in humans and in mice have established a link between being overweight or obese and oxidative stress and sperm DNA damages [45], [46]. In the present study, significantly higher levels of SOD2 protein have been observed in seminal plasma after the dietary program. Moreover an important decrease of carbonylated proteins has been evidenced, indicating an increased quality of the seminal proteome. Whether an improvement of the balance between oxidative/anti-oxidative substances in the seminal plasma due to the dietary program remains to be determined in future studies.

Moreover, an improvement of the testosterone/oestradiol ratio was observed in parallel with decreased abdominal fat. Central adiposity has been associated with lower levels of testosterone [47], and oestradiol levels positively correlate with visceral fat, but not with subcutaneous, adipose tissue. Therefore, visceral adipose tissue strongly correlates with testosterone/oestradiol ratio [48]. Based on an experimental mouse model, obesity, inflammation and visceral fat concentrations of aromatase appear to be linked [49]. The aromatization process, leading to the conversion of testosterone into oestradiol, is mediated by visceral fat inflammation. Obesity is associated with elevated of pro-inflammatory molecules that are known to induce the aromatase gene transcription and the aromatase activity [50], [51]

## Conclusion

In conclusion, even a limited number of patients, these original findings suggest that abdominal fat loss may improve sperm DNA integrity, blood hormonal profile and pregnancy outcome. Oxidative stress should be involved in this phenomenon since an increase of SOD2 level and a decrease of oxidatively damaged proteins have been evidenced in seminal plasma of these patients after the dietary program.

Further prospective controlled interventional studies are needed to observe the effect of loosing abdominal fat on sperm quality in a larger cohort of infertile men with high sperm fragmentation and imbalance between oxidative and anti-oxidative substances in semen. If these results were confirmed, future prevention of subfertility should target abdominal fat and oxidative stress mainly in men with high sperm fragmentation.

Further studies are also needed to understand mechanisms and the effect on fertility outcome.
